# The impact of ERAS protocol on laparoscopic sleeve gastrectomy and one anastomosis gastric bypass (OAGB): analysis of length of stay (LOS), complications, and readmission

**DOI:** 10.1007/s13304-025-02152-x

**Published:** 2025-05-19

**Authors:** Samar Shalaby, Riadh Salem, Liz Ward, Caroline Fletcher, Bruno Sgromo

**Affiliations:** 1 Buckinghamshire NHS Trust, Buckingham, HP21 8AL UK; 2 Oxford University Hospitals NHS Foundation, Oxford, UK; 3 Department of Dietetics, Wiltshire Health & Care, Chippenham, England SN152AJ UK; 4 Nuffield Department of Surgery, Oxford University Hospitals, Oxford, UK

**Keywords:** ERAS Protocol, Bariatric, Sleeve gastrectomy, One anastomosis gastric bypass

## Abstract

Enhanced Recovery After Surgery (ERAS) protocols have transformed care in bariatric surgery, leading to faster recovery and shorter hospital stay. This study evaluates the impact of ERAS protocols on laparoscopic sleeve gastrectomy (SG) and one anastomosis gastric bypass (OAGB), focusing on key elements such as length of stay (LOS), postoperative complications, and readmission rates. A retrospective data analysis was conducted on patients who underwent SG or OAGB under an ERAS protocol from 2021 to 2024 evaluating a total of 181 patients in both groups. Data on LOS, complications, and readmissions were collected and compared between the two groups. The results indicate that the implementation of ERAS protocols significantly reduces LOS for both SG and OAGB patients, with average reductions of 1.2 days and 1.5 days, respectively. Additionally, the complication rates and readmission rates were found to be lower in patients undergoing these procedures with ERAS protocols compared to historical controls (Kehlet in Br J Anaesth 78:606–617, 1997; Stenberg et al. in World J Surg 46:729–751, 2022). This study highlights the effectiveness of ERAS in optimising recovery and reducing hospital costs in bariatric surgery. In the UK, only about 50% of bariatric units use a formal Enhanced Recovery After Surgery (ERAS) pathway. Using these protocols is important for improving patient outcomes by reducing complications and shortening hospital stays (Stenberg et al. in World J Surg 46:729–751, 2022).

**Supplementary Information:**

The online version contains supplementary material available at 10.1007/s13304-025-02152-x.

## Introduction

Bariatric surgery has become a widespread and essential procedure globally for the treatment of morbid obesity. Enhanced Recovery After Surgery (ERAS) protocols, conceptualised in the late 1990s by H. Kehlet for colorectal surgery, have gained widespread acceptance across various surgical disciplines due to their association with improved patient outcomes and reduced hospital length of stay [1, 2]. ERAS protocols focus on optimising preoperative preparation, minimising intraoperative trauma, and promoting early postoperative rehabilitation. Extensive literature highlights several advantages of ERAS, such as reduced hospital length of stay, improved patient outcomes, decreased postoperative complications, cost-effectiveness, and enhanced patient satisfaction. However, bariatric surgery poses unique challenges related to both the patients and the specific nature of the operations. While early discharge offers significant benefits to healthcare systems, any delay in diagnosing postoperative complications can result in severe consequences. This study aims to assess the safety profile of an aggressive ERAS protocol following sleeve gastrectomy (SG) and one anastomosis gastric bypass (OAGB). Additionally, it seeks to identify and analyse the differences in outcomes between these two bariatric procedures. In the UK, it is reported that only about 50% of bariatric units have a formal Enhanced Recovery After Surgery (ERAS) pathway in use. The implementation of these protocols is crucial for optimising patient outcomes, including reducing postoperative complications and shortening the length of hospital stays [2].

## Methods

All patients (*n* = 181) in this study were referred for considerations of bariatric surgery following a non-surgical weight loss management. A specialist assessment was performed for every patient by dietitian and surgeon, ad hoc psychological assessment was performed, and every patient underwent a preoperative assessment with specialist nurse and/or anaesthetist.

Each patient’s case was discussed at a formalised MDT and bariatric surgery was offered in accordance to NICE guidance.

The choice of the operation was decided in accordance with the patient's choice, dietitian’s, and surgeon’s advice.

### Surgical technique

All the cases were performed laparoscopically with 3 operative ports and a Nathanson liver retractor. An anterior repair was performed if an hiatus hernias was found during the dissection. The majority of the operations was performed using a LigaSure™ Maryland Jaw Laparoscopic Sealer/Divider but in few cases with the Thunderbeat™ or CoolSeal™ Trinity™.

### Sleeve gastrectomy [3–5]

The sleeve gastrectomies were performed using the EndoGIA (Medtronic) stapler with 60mm cartridges (3 purple and 2 tan) starting 4–6 cm from pylorus. After firing the first cartridge and ensuring the angulus was cleared to avoid narrowing, a 38Fr orogastric tube was advanced into the antrum. The specimen was extracted through a port site.

### One anastomosis gastric bypass [6–11])

A 15cm gastric pouch size was fashioned using a with the use of Endo GIA™ Universal Stapler 45(× 1) and 60mm cartridges (2 purple and 2 tan), with antecolic reconstruction and a biliary limb tailored in length based on BMI (150 cm for BMI > 50, and 100 cm for BMI < 50). The anastomosis was performed using Endo GIA 30mm (purple) and v-lock sutures in a two-layer closure of the enterotomy, facilitated over a 38Fr orogastric tube.

Before finishing each operation the blood pressure was raised to over 125 systolic for 5 min before any staple line bleeding was excluded. Bleeding from the staple line was managed with Endo Clip™, Surgicel® Powder Hemostat and Veriset™ Hemostatic Patch as necessary.

Bupivacaine 0.25% 60 mls was used at the end of each operation in the form of port infiltration, bilateral TAP blocks, and intra-peritoneal spray.

All patients were managed postoperatively in a Level II (HDU) facility according to a standardised ERAS protocol (attached) by experienced nurses for the first postoperative night.

Data from the operations were collected and recorded in both the national registry and the hospital's local database (Manor), providing comprehensive and reliable datasets for analysis. The two databases were cross checked for the analysis.

### ERAS protocol

The "Nuffield Health Bariatric Surgery Integrated Care Pathway”, developed originally by the authors LW, CF, and BS in 2009 and regularly modified, is a comprehensive document outlining the standardised protocol for the management of patients undergoing bariatric surgery. Attachment 1.

### Discharge criteria

For patient discharge, the following criteria must be satisfied:

- The NEWS score was the tracking system used and a score below 3 was used as threshold [13].
- Pain management: Effective pain control achieved with oral analgesics.
- Nutritional intake: Adequate oral fluid and protein intake.
- Mobility: Independence in mobility and personal care.
- Completion of discharge checklist: Includes medication instructions and follow-up care plans.

The pathway emphasises rigorous documentation practices. Variations from the prescribed care are recorded, additional care plans are implemented when necessary, and all entries are made in black ink to comply with legal standards. Practitioners are required to document their actions and provide their signatures to ensure accountability.

Baseline, perioperative, and outcome categorical variables were compared using chi-square test, and non-parametric continuous variables were compared using a two-sample t-test. To account for differences in baseline variables between SG and OAGB which could affect the primary outcome, a propensity matched analysis was performed using SPSS. Firstly, a propensity score was generated using logistic regression for baseline variables including age, sex, and BMI. Operative approach was the dependent variable. Two study groups were then selected using “nearest neighbour” matching. SPSS version 24 (IBM) was used.

## Results

Between January 2021 and December 2024, a total of 181 operations (83 OAGB and 98 SG) were performed and included in this study.

The two groups’ demographics and baseline characteristics are described in Table 1.

**Table 1:** Groups characteristics

Parameter	OAGB	SG
Total number of patients	84	97
Average age (year)	41.7	42.33
Average range (year)	20–70	20–73
Average BMI (kg/m^2^)	47.76	40.45
BMI range (kg/m^2^)	33.0–65.0	33.2–64.0
Gender distribution (F:M)	71:13	80:17

### Comorbidities analysis

This analysis compares the prevalence of various associated diseases between patients undergoing sleeve gastrectomy (SG) and one anastomosis gastric bypass (OAGB). Diseases analysed include hypertension, ischaemic heart disease (IHD), type 2 diabetes mellitus (T2DM), polycystic ovary syndrome (PCOS), obstructive sleep apnoea (OSA), depression and anxiety, and hypothyroidism.
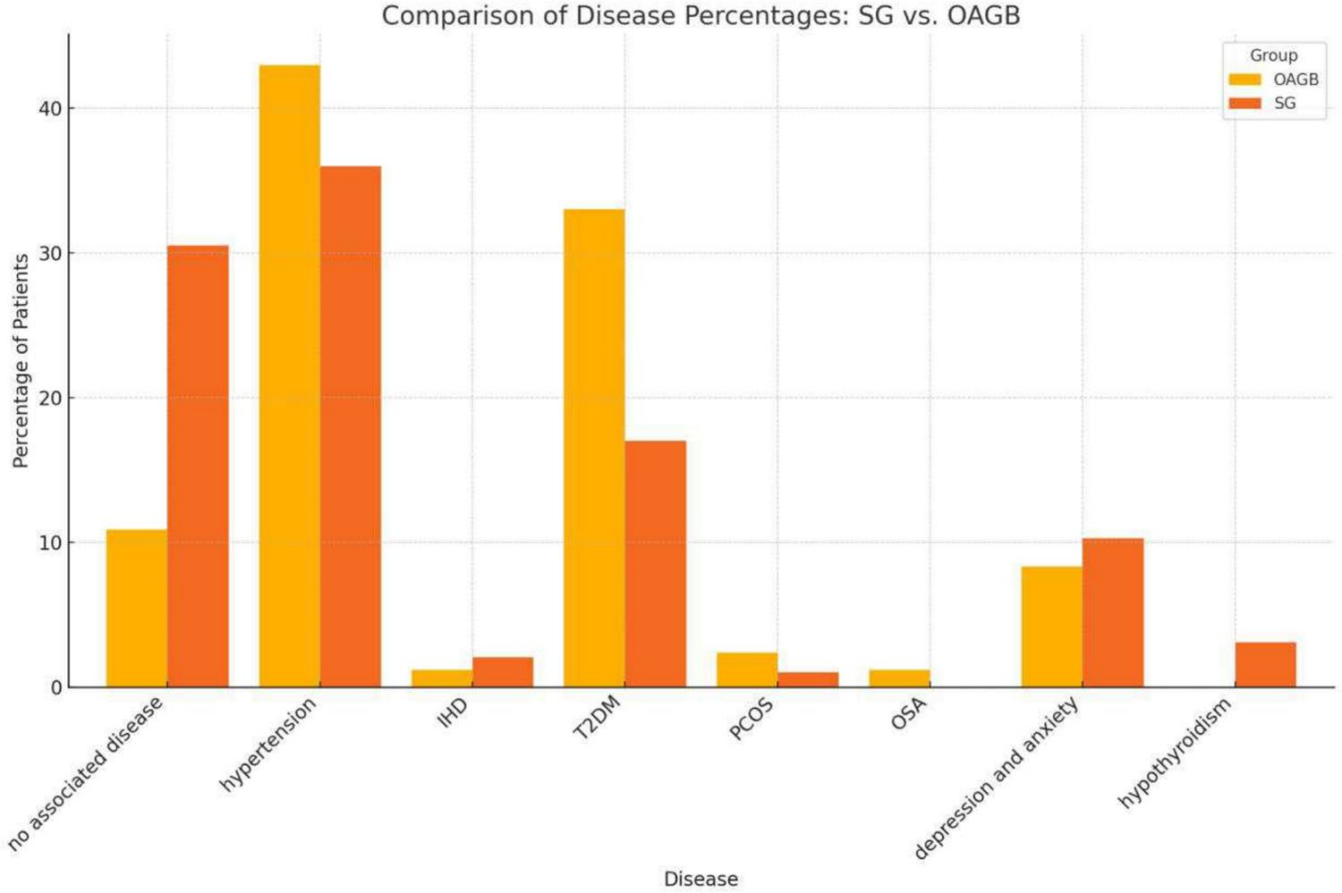


Chi-square test results showed statistical significance in T2DM (*P* value = 0.0201).

No other statistical significance differences were noted in the rest of the comorbidities.
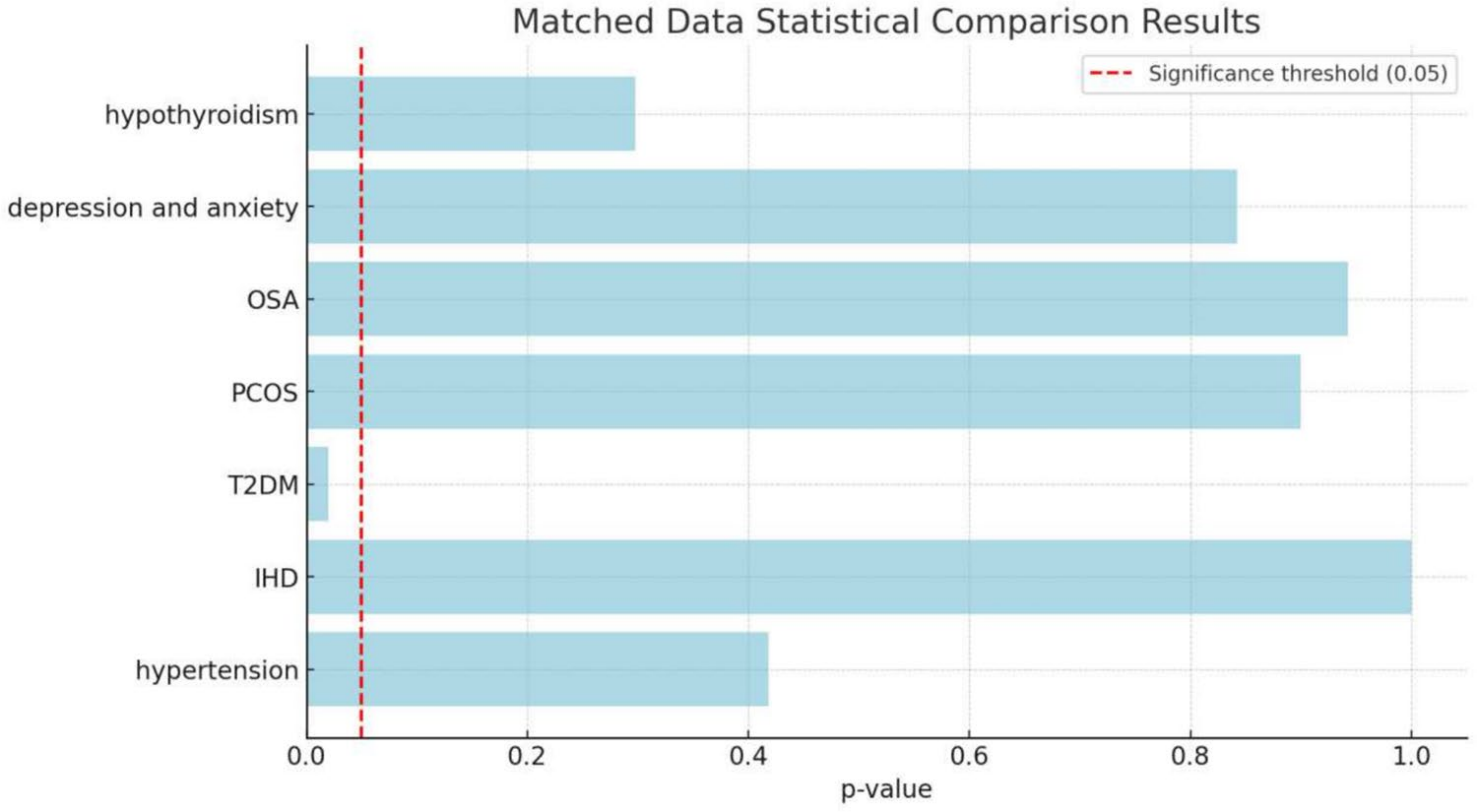


### Length of stay (LOS)

A discharge home on the first postoperative day was achieved in 145/181 (80%) of the patients. In the OAGB group in 64/83 (77%) and in the SG group in 81/98 (82%). The difference in LOS between the two groups was not statistically significant (*P* value = 0.1029).

35 of 181 (19%) patients stayed in the hospital for more than 1 night, 17/98 (17%) patients in the SG group and 18/84 (21%) in the OAGB group (Table 2).

**Table 2:** Causes of failed discharge on day 1

Causes for failed discharge on day 1	SG (17)	OAGB (18)
Nausea	10	11
Pain	4	3
Social circumstances	3	4


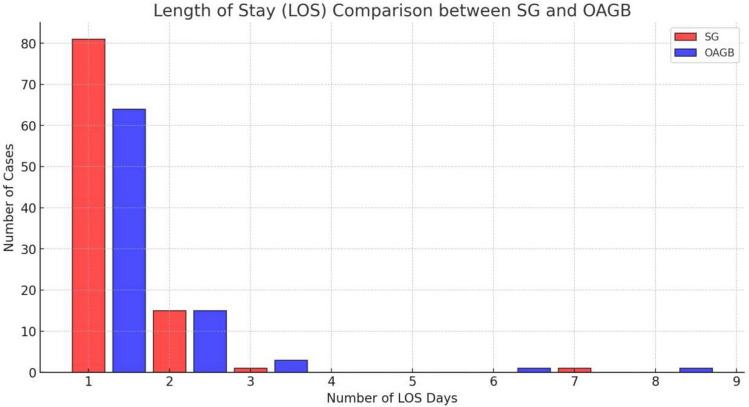


### Complications, reoperations, and readmission.

In the OAGB group, two patients suffered a Clavien–Dindo > 3 (2/84 2.3%) [14].

One patient experienced postoperative bleeding suspected due to a drop in Hg and tachycardia and confirmed on CT scan as a peri-gastric haematoma. This was treated with a laparoscopic washout and drainage and was discharged home on day 6 after the index operation.

A second patient was initially discharged home on day one postoperatively but readmitted on day 14 with intra-abdominal sepsis. A CT scan showed a peri-gastric collection. The patient was taken back to the operating theatre for a laparoscopic laparoscopy and found to have a pinhole defect in the gastrojejunostomy with peri-anastomotic walled-off collection. The wash out drainage led to a resolution of the sepsis and the patient was subsequently discharged home on day 7 after the reoperation.

In the SG group, one patient was taken back to theatre on the first postoperative day due to bleeding (1/98–1%). This was suspected due to a drop in Hb despite normal observations. Following the laparoscopic washout and drainage, the patient was discharged home on day 6 after the index operation.

## Discussion

This study aims to evaluate the impact of the Enhanced Recovery After Surgery (ERAS) protocol on bariatric patients undergoing sleeve gastrectomy (SG) and one anastomosis gastric bypass (OAGB) in terms of length of stay (LOS), complication rates, and readmission rates.

During the same period number,13 patients underwent a Roux en Y Gastric Bypass, we decided not to include these cases in study due to the small number of operations done, too few for a meaningful analysis despite no complications occurred in this group. A RYGB was preferred to the SG and the OAGB in case of severe GORD symptoms or the presence of Barrett’s oesophagus.

The demographic analysis of both datasets showed that the mean age of patients undergoing SG and OAGB was comparable, with no significant differences. However, a notable difference was observed in the Body Mass Index (BMI) of patients in the not matched dataset, where the BMI for OAGB patients was significantly higher than that for SG patients (47.8 vs 40.4, *p* < 0.01). This suggests that OAGB might be preferred for patients with higher BMI, due to its efficacy in managing severe obesity and associated comorbidities [15].

The implementation of the ERAS protocol demonstrated its effectiveness in achieving a length of stay of 1 night in hospital for both surgical procedures in 82% and 77% of the patients in the SG and OAGB groups, respectively. This outcome was in keeping with our expectations and considering that a number of patients stayed for longer than one night for non-clinical reasons we can confidently say that a 1-night in-hospital stay is safely and consistently achieved in over 80% of all patients regardless of the operation or comorbidities [12].

The complication rates were low and within the expected range across both datasets, reflecting the safety and effectiveness of bariatric surgery and the ERAS protocol.

The ERAS protocol facilitated the work of the nursing team, providing consistency in the delivered care and allowing an early diagnosis of abnormal physiology. This happened in all cases but in one of the patients with complications where the NEWS score was 0 but the blood test showed a drop in Hgb.

This study confirms the paramount importance of early re-interventions in case of suspected complications as shown by the rapid recovery after a reoperation.

The readmission rate for both procedures was low and within published results. As demonstrated by the only patient who was readmitted and reoperated, an effective safety net to capture patients in difficulties is essential. Anastomotic leaks after OAGBs can be dramatic complications, luckily in our patient, the leak was contained, and no peritonitis was present at the time of the reoperation. The outcome would have been different if a delay in the reoperation had occurred.

To further reduce the LOS alternative medical strategies for postoperative nausea and vomiting could be implemented as well as pain relieve medications.

## Conclusion

In conclusion, our Enhanced Recovery After Surgery (ERAS) protocol has proven to be highly effective in improving outcomes for bariatric patients undergoing sleeve gastrectomy and one anastomosis gastric bypass. The protocol successfully allows a short length of stay and maintains low complication and readmission rates, confirming its value in bariatric surgical practice. Further large-scale, multicentre studies are needed to validate these findings and to explore the full potential of the ERAS protocol in various patient populations.

## Supplementary Information

Below is the link to the electronic supplementary material.

Supplementary file1 (PDF 443 KB)

## Data Availability

Not applicable.
