# Supplementary material for: The impact of ERAS protocol on laparoscopic sleeve gastrectomy and one anastomosis gastric bypass (OAGB): analysis of length of stay (LOS), complications, and readmission
Source: Updates Surg. 2025 May 19;77(7):2107–12. doi: 10.1007/s13304-025-02152-x (PMC12540629; doi:10.1007/s13304-025-02152-x)
Supplement: Supplementary file 1 — Supplementary file1 (PDF 443 KB) [file 13304_2025_2152_MOESM1_ESM.pdf]

Patient Label

BARIATRIC SURGERY

Consultant:	
Planned Operation:	
Admission Date:	
Discharge Date:	

This Care Pathway should be added to the Nuffield Health Care Record(NHCR). All variance occurred and why, and actions taken and outcomes should be documented ion the NHCR – Multidisciplinary Evaluation& Variance area.

TO BE COMPLETED ON DAY OF ADMISSION		
Patient's weight: .... kg	Patient's height: .... M	Date: ....
Patient's BMI:		

THE EVIDENCE AND GUIDELINES UNDERPINNING THIS PATHWAY ARE:

Medication guidelines following bariatric surgery

ANTIEMETICS		
1st line	Metoclopramide 10mg, oral or IV	prescribed TDS regularly for 3/7
	Prochlorperazine (Buccastem) 3-6mg, buccal	prescribed BD regularly for 3/7
2nd line	Ondansetron 4-8 mg, oral or IV (not suitable for patients with Q-T prolongation on ECG	prescribed PRN
3rd line	Cyclizine 50mg, oral or IV	prescribed PRN (maximum TDS)

Post-operative analgesics	
IV Fentanyl rescue	Administered in recovery
Paracetamol	prescribed QDS regularly
Ibuprofen, 400mg, oral	prescribed TDS regularly, if appropriate
Tramadol 50-100mg, QDS, oral	prescribed PRN – caution as may cause constipation
Morphine sulphate, 10mg/5ml, oral solution, 10-20mg, 2-4 hourly OR Oxycodone, 5mg/5ml, oral solution, 5-10mg, 2-4 hourly	prescribed PRN – caution as may cause constipation

Morphine PCA should be avoided in these patients as it can cause respiratory depression and exacerbation of pre-existing breathing problems.

Proton-pump inhibitors	
Lansoprazole FasTabs, 30mg, orally	Prescribed OD regularly from day of surgery
**If Lansoprazole contraindicated**: Ranitidine (dispersible) 300mg orally OD	N.B. moderate to severe risk of renal impairment

Post-operative Dalteparin dosing guidelines

The licensed dose for high risk surgical patients is 5,000 units once daily, however there is some evidence to suggest dose banding based on weight (see below) may provide more effective prophylaxis and should be considered in patients at the extremes of body weight.

On the day of surgery the dose may be split – check the post-operative note for instructions.

Weight (kg)	Dose (units)
40-120	5,000 once daily
121-150	7,500 once daily
More than 150	5,000 twice daily

EXTENDED VTE PROPHYLAXIS

It has been acknowledged that patients undergoing bariatric surgery are at high risk for developing a post-operative venous thromboembolism. Therefore patients undergoing sleeve gastrectomy or gastric bypass surgery should be **discharged on Dalteparin**. This will extend to <u>28 days post-surgery</u>.

THE EVIDENCE AND GUIDELINES UNDERPINNING THIS PATHWAY ARE:

ORAL INTAKE PROGRESSION	
Day of surgery	Sips of clear fluids orally up to **500mls in 24 hours**
Post-op day 1	Free fluids orally with protein shots (Altraplan/Fresubin protein energy) 30ml every hour for 10 hours
Post-op day 2 onwards	Free fluids orally with protein shots (Altraplan/Fresubin protein energy) 30ml every hour for 10 hours

Protein-rich free fluids should be consumed first to provide the required 60g protein, followed by other free fluids listed below to achieve 2000ml total fluid intake.

PROTEIN-RICH FLUIDS INCLUDE:	
Nualtra Altraplen Protein drink (200ml bottle)	
~~Fresubin Protein Energy (200ml bottle)~~	
Thin, Sieved soup	
Milk (skimmed or semi-skimmed)	
**DO NOT give the following protein-rich fluids:**	
Ensure drinks	
Fortisip	
Fortijuce	
**Free fluids include:**	
Tea / coffee without sugar	Unsweetened pure fruit juice (diluted with water)
Still water	Low calorie hot chocolate, Ovaltine or Horlicks is included if made with milk but not if made with water or a non-low calorie variety.
Low calorie or no added sugar squash	
**DO NOT give the following free fluids:**	
Sweetened pure fruit juice	Unsweetened pure fruit juice not diluted with water
All fizzy/carbonated drinks	Shop bought smoothies
Vegetable juice	Probiotic drinks

BARIATRIC DIABETES SYSTEMS OF PRACTICE PROTOCOL
Diabetes medication advice to be provided by Mr Sgromo on an individual patient basis.

SUPPLEMENTARY OXYGEN VIA CPAP (CONTINUOUS POSITIVE AIRWAY PRESSURE)
If the patient usually uses a CPAP machine at night for sleep apnoea, and they require supplementary O_2, we should use an adaptor to the CPAP machine to supply the O2.

INTEGRATED CARE PATHWAY

Guidance for use

• This care pathway is a guideline for best multi-disciplinary care for the patient. It is *not* a substitute for clinical judgement and expertise.
• Decisions regarding care remain at the discretion of the clinician. Use your professional judgement to decide if actions and timings are appropriate for the patient.
• If you are going to write in this care pathway you must document your Name, Designation and give a sample signature and initials on the next page.
• To meet the legal requirement, documentation should be accurate, comprehensive and written in black ink.
• Additional care plans are to be used for care not documented on this pathway (e.g. Fluid Balance charts).
• Sign only for care that YOU have carried out or outcomes that have been met.
• When completing the pathway, in the relevant shift column, insert:<br>– Your **initials** if the outcome/plan has been met,<br>– **V** (for variance) if the outcome has not been met,<br>– **N/A** if the outcome/plan is not applicable to that patient.
• Practitioners remain free to exercise their own professional judgement; however, where a clinical decision would result in a variation from the treatment and care set out in this pathway must be recorded as a variance (V).
• Variances must be documented in the relevant box. Document the action you will take to try to bring the patient back onto the pathway. Insert extra pages as necessary.
• If the outcome/plan in the care pathway does not give enough information to accurately document the care provided, you must use the NHCR pages to add additional information. These notes should always be dated and signed. Insert extra pages as necessary.

Abbreviations used in this document

AM	Morning
ANTT	Aseptic Non-Touch Technique
BD	Twice Daily
BMI	Body Mass Index
CPAP	Continuous Positive Airway Pressure
g	Gram(s)
GI	Gastrointestinal
IM	Intramuscular
IV	Intravenous
kg	Kilogram(s)
L	litres
M	Metre(s)
mg	Milligram(s)
ml	Millilitre(s)

NB	Nota bene
OD	Once daily
PCA	Patient controlled analgesia
PM	Afternoon
PO	Orally
PPI	Proton-pump inhibitor
PRN	As required
QDS	Four times daily
RYGB	Roux-en-Y gastric bypass
SC	subcutaneous
TDS	Three times daily
T&T	Track and Trigger
TTO	Tablets to Take Out
VIP	Visual Infusion Phlebitis
VTE	Venous Thromboembolism

ALL PERSONNEL COMPLETING THE PATHWAY PLEASE SIGN BELOW:

Print Name	Designation	Signature	Initials

Nuffield Health
FOR THE LOVE OF LIFE®

Day of Surgery:

Date:	Room:
Time returned to ward:	

BARIATRIC SURGERY CARE PATHWAY

Plan/Goal: Recover from anaesthetic, observations stable, pain and nausea are controlled

Observations and Respiratory:	AM	PM	Night
Observations and Track & Trigger score completed ½ hourly for first 4 hours, then hourly for 4 hours, frequency of observations dictated by Track & Trigger score.			
Oxygen in place as prescribed and saturations maintained in accordance with prescribed range			
If patient usually uses a CPAP machine and is drowsy/sleepy – Oxygen-enriched CPAP administered			
Deep breathing promoted, patient able to deep breathe and cough			
**Nutrition/Intake:**			
IV maintenance fluids administered as prescribed (usually 2L in the first 24 hours)			
Patient is tolerating sips of water orally, 30mls/h (max 500mls/24h)			
Patient has been prescribed and received Lansoprazole FasTabs 30mg once daily unless contraindicated (see table on page 2)			
Anti-emetics given regularly as prescribed			
**Elimination/Output:**			
Patient has no complaints of nausea and vomiting			
Patient has passed urine, documented on fluid balance chart			
**Pain:**			
Prescribed analgesics given orally/IV			
Pain assessment recorded at each set of observations (aim pain score 0-4/10)			
**Wound/Drains:**			
Wound observed when observations recorded – no bleeding/signs of infection			
Peripheral line (VIP) score = 0			
**Thromboprophylaxis:**			
VTE risk assessment completed and appropriate prophylaxis prescribed			
Anti-embolic stockings or Flowtron boots and VTE prophylaxis administered as prescribed			
**Mobility:**			
Pressure areas checked and actions taken accordingly (document actions on the RSKIN form)			
Patient is in head-up position in bed			
Patient has appropriate footwear on for mobilising (e.g. ward gripper socks/slippers)			
Patient has sat out of bed for 30-60 minutes within 4 hours of surgery			
Patient has had a walk (100m) within 4 hours of surgery			
**Hygiene:**			
Patient assisted to identify and meet their personal hygiene needs			
Patient prompted to self-administer mouth care			
**Education:**			
Patient reminded to complete their post-operative plan booklet, assist if necessary			

Post-operative Day 1

Nuffield Health
FOR THE LOVE OF LIFE®

Post-operative Day: 1

Date:	Room:

BARIATRIC SURGERY CARE PATHWAY

Plan/Goal: Sit out of bed, mobilise with assistance, pain and nausea controlled, aim for discharge if suitable for discharge & criteria met

**Observations and Respiratory:**	**AM**	**PM**	**Night**
Observations and Track & Trigger score completed 4 hourly. Actions taken as per T&T escalation pathway (document on reviews and evaluations page)			
Oxygen saturations ≥ prescribed target without supplementary oxygen			
Deep breathing promoted, patient able to deep breathe and cough			
**Nutrition/Intake:**			
IV maintenance fluids administered as prescribed			
IV fluids discontinued if oral intake >500ml by midday			
Patient drinking free fluids (aim for 1000ml-1500ml)			
Patient has had 10 Protein shots (Altraplen or Fresubin) (aim for 30ml every hour for 10 hours)<br>AM ☐ ☐ ☐ ☐ ☐ ☐ ☐ ☐ ☐ ☐<br>PM ☐ ☐ ☐ ☐ ☐ ☐ ☐ ☐ ☐ ☐<br>Night ☐ ☐ ☐ ☐ ☐ ☐ ☐ ☐ ☐ ☐			
Patient has been prescribed and received Lansoprazole FasTabs 30mg once daily unless contraindicated (see information on page 2)			
**Elimination/Output:**			
Patient has no complaints of nausea or vomiting			
Anti-emetics given regularly as prescribed Patient			
passing good volumes of urine			
Patient has opened their bowels, if not consider sodium docusate			
**Pain:**			
Pain assessment recorded at each set of observations (aim pain score 0-1/3)			
Prescribed analgesics given orally/IV			
**Wound/Drains:**			
Wound observed – no bleeding/signs of infection. Dressing changed if required			
Lines/Prevention of infection: (refer to ANTT and line care guidelines) Peripheral			
line (VIP) = 0, documented electronically. Line removed if indicated Bloods taken			
and results checked (FBC, U+E, Creat, CRP, Phosphate, Mg) **Thromboprophylaxis:**			
Anti-embolic stockings or Flowtron boots and VTE prophylaxis administered as prescribed			
**Mobility:**			
Pressure areas checked and actions taken accordingly (document actions on the RSKIN form)			
Patient has sat in the chair for 2-3 hours on 3 separate occasions<br>AM ☐ ☐ ☐<br>PM ☐ ☐ ☐<br>Night ☐ ☐ ☐			
Patient has mobilised, aim 3 x length of ward<br>AM ☐ ☐ ☐<br>PM ☐ ☐ ☐<br>Night ☐ ☐ ☐			

Post-operative Day: 1

Date:	Room:

Hygiene:	AM	PM	Night
Patient assisted to meet their own personal care needs			
TEDS changed			
**Education:**			
Patient is provided with a copy of the post op dietary information (if they do not have a copy with them)			
**Aim for discharge today:** (if the criteria below are met, why is the patient still in hospital?)	AM	PM	Night
Patient identified for discharge in medical notes	Yes		No
Effective pain control with oral analgesics (within acceptable limits for the patient)			
Nausea under control, no vomiting			
Patient tolerating 1000ml - 1500ml oral fluids including protein-rich fluids			
Independently mobile, able to get self out of bed and on/off toilet			
Discharge checklist completed on page 17			

Patients progress review on ward round		
Plan of progress:		
Please document all additional information that is not a variance to the planned care pathway in the space below (sign and print name against each entry) (additional sheets may be added)		
Date /time:	Post-op day	
